# Endogenous gene selection for relative quantification PCR and IL6 transcript levels in the PBMC’s of severe and non-severe dengue cases

**DOI:** 10.1186/s13104-018-3620-2

**Published:** 2018-08-02

**Authors:** Vigneshwari Easwar Kumar, Cleetus Cherupanakkal, Minna Catherine, Tamilarasu Kadhiravan, Narayanan Parameswaran, Soundravally Rajendiran, Agieshkumar Balakrishna Pillai

**Affiliations:** 1 Central Inter-Disciplinary Research Facility, Sri Balaji Vidyapeeth (Deemed to be University), Puducherry, 607402 India; 2 Department of Biochemistry, Jawaharlal Institute of Postgraduate Medical Education and Research, Puducherry, India; 3 Department of Medicine, Jawaharlal Institute of Postgraduate Medical Education and Research, Puducherry, India; 4 Department of Pediatrics, Jawaharlal Institute of, Postgraduate Medical Education and Research, Puducherry, India

**Keywords:** Dengue severity, Endogenous gene selection, qPCR, geNorm, Normfinder, PBMC

## Abstract

**Objectives:**

Dengue viral infection ranges from dengue fever to dengue haemorrhagic fever and lethal dengue shock syndrome. Currently no means are available to monitor the progression of disease. Real time PCR based gene expression analyses are used to find potential molecular markers for effective prediction of dengue clinical outcome. The accuracy of qPCR analysis is strongly dependent on transcript normalization using stably expressed endogenous genes, which if selected imprecisely can lead to misinterpreted results. We aimed to determine the best fit for endogenous gene among six genes namely COX, ACTB, GAPDH, HMBS, HPRT and B2M for dengue viral infection cases. Gene stability was inferred from qPCR data by normalizing with two algorithms geNorm and Normfinder and the rankings generated were validated by gene expression analysis against target gene IL-6.

**Results:**

Both the algorithms showed ACTB, HPRT, GAPDH as most stable genes. Normalizing with the stable genes revealed a significant fold change (p < .05) in IL-6 levels of .32, .52, .69, and .62 in non-dengue febrile illness, non severe, severe and All Dengue groups respectively compared to healthy controls. based on our study, we suggest ACTB with HPRT/GAPDH combination for normalization in qPCR for precise quantification of transcripts in dengue infected studies.

**Electronic supplementary material:**

The online version of this article (10.1186/s13104-018-3620-2) contains supplementary material, which is available to authorized users.

## Introduction

Dengue infection caused by the dengue virus (DENV) of the Flaviviridae family is a mosquito borne viral disease affecting over 100 million lives annually worldwide [1]. It occurs in four antigenically distinct serotypes (DENV-1 to DENV-4) and is transmitted by the mosquito vector *Aedes aegypti* (*Ae*. *Aegypti*). Patients with dengue show a wide clinical spectrum ranging from no symptoms, or characteristic dengue fever (DF) and dengue haemorrhagic fever (DHF) which often causes plasma leakage resulting in dengue shock syndrome (DSS) or severe dengue (SD) [2]. DF and DHF patients display a very similar clinical picture during acute febrile illness stage but at defervescence (after 4–7 days of the beginning of the symptoms) circulatory disturbance is observed in DHF patients [3]. If prognosis of the disease and its progress from DF to SD is identified early, hospitalization costs of patients displaying only DF can be reduced significantly and appropriate medical assistance can be provided to high risk patients. Molecular biology tests are now being developed to help differentiate patients with DF from those with DF culminating in DHF as hematological and biochemical tests fail to accurately correlate the clinical outcome [2]. Recent studies reported some of the host transcripts that are specifically up or down regulated in severe cases compared to non-severe cases which include genes encoding for macrophage cytokines such as IL-4, IL-6, IL-8, IL-10, IL-12, TNFα, and IFNγ [3].

Presently, real time or quantitative real-time polymerase chain reaction (qPCR) is widely used molecular biology assay for gene expression analysis. Due to minimal sample requirement, specificity and sensitivity, qPCR has found use in faster detection of diseases. Though advantages in terms of accuracy and automation abound, the interpretation of the generated qPCR data is challenging due to the increased chance of errors given the various stages of sample preparation and processing. These include differences in starting sample quantities during RNA extraction and reverse transcription to cDNA, its quality and storage and also experimental design, primer selection and statistical analysis [5, 6]. Therefore, normalization of qPCR data against an endogenous reference gene is required to account for these differences. Among biological, exogenous and genetic normalization, genetic normalization is most frequently used [7]. Genetic normalization is performed by using endogenous reference genes. An endogenous reference gene commonly called “housekeeping gene” (HKG) is any gene that is stably expressed under all developmental and experimental conditions such as genes associated with metabolism. But it has been observed that no single gene can satisfy this condition which makes choosing a set of genes as reference for normalization empirical. Thus, imprecise selection of reference genes can lead to misinterpretation of results and calls attention for proper validation of these genes. To determine HKG stability, algorithms such as geNorm, Normfinder and Bestkeeper have been developed for identifying the best-fit reference genes for one’s experimental condition. In the present study best endogenous gene among COX, ACTB, GAPDH, HMBS, HPRT and B2M during dengue fever. These genes were chosen as candidate reference genes based on their demonstrated performance as reference genes in previous studies on human PBMC [8, 9]. GeNorm and Normfinder were used to rank the reference genes based on their stability. In order to validate the ranking generated by the programs, we performed gene expression analysis by normalizing to the geometric mean of best fit and poorly fit reference genes against a chosen target gene IL-6. IL-6 was chosen as the target gene as a marked upregulation of the gene has been reported in dengue cases [4].

## Main text

### Methods

#### Subjects

The study subjects were recruited from Jawaharlal Institute of Post Graduate Medical Education and Research (JIPMER) hospital, Puducherry, India and blood samples from patients belonging to Puducherry and Tamilnadu, India were collected during the dengue fever outbreak in the year of 2012–2014. The prospective cohort study consists of 34 dengue patients (count includes both Severe and Non Severe Dengue), 20 other febrile illness (OFI) subjects and 16 healthy controls. After taking written informed consent, 3 mL of blood was collected within 24 h of admission (Febrile period).

#### PBMC isolation, RNA extraction and cDNA synthesis

PBMCs were separated from whole blood obtained from Healthy controls, OFI and dengue cases using HiSep LSM 1077 (Himedia, Mumbai, India), washed with PBS twice and stored in 1 mL RNAiso Plus reagent (Takara Bio Inc., Shiga, Japan) and kept at − 80° as described in our earlier studies [10]. Total RNA was extracted using RNA easy minikit (Qiagen, GmbH, Hilden, Germany) based on the manufacturer’s protocol. Any endogenous DNA was removed by treating with RNase free DNAse set (Qiagen, GmbH, Hilden, Germany) supplied by the manufacturer. NanoDrop spectrophotometer (Thermo Scientific, Waltman, MA, USA) was used for assessing purity and concentration of RNA. 1 μg of RNA was used to synthesize complementary DNA (cDNA) using the high capacity cDNA reverse transcription Kit with RNase inhibitor (Applied Biosystems, Foster City, CA, USA). The cDNA thus obtained was used for qPCR data analysis of the six candidate reference genes and selected target gene in a 40 cycle PCR.

#### qPCR analysis

The primers of the endogenous reference genes and target gene were used based on previous studies [9, 10, 17, 20] and are described in Additional file 1: Table S1. All reactions were performed in duplicates with a standard run protocol of initial denaturation at 95° for 30 s followed by 40 cycles of denaturation (95° for 5 s) and a combined annealing and extension (60° for 30 s).

### Results

#### Expression levels and statistical analysis

The highest Cq value among the six candidate reference genes was recorded in B2M at 11.55 while the lowest value was recorded in HPRT at 29.18. Figures of gel images showing single bands corresponding to selected candidate reference genes is given in Additional file 2: Figure S1, Additional file 3: Figure S2, Additional file 4: Figure S3, Additional file 5: Figure S4 and Additional file 6: Figure S5. The mean Cq values of the genes for each group (healthy control, OFI and dengue groups) and statistical analysis is shown in Table 1. Statistics was computed and all Cq values obtained were found to follow normal distribution using Shapiro–Wilk’s test.

**Table 1:** Result of statistical analysis of Cq values of selected candidate reference genes and target genes

Gene	Type				
	HC	OFI	NSD	SD	ALL DENGUE
COX	24.14 ± 1.15 (N = 10) (p = .674)	24.47 ± 1.80 (N = 10) (p = .996)	25.67 ± 1.62 (N = 10) (p = .209)	25.32 ± 1.517 (N = 10) (p = .733)	25.49 ± 1.540 (N = 20) (p = .120)
ACTB	18.97 ± .862 (N = 10) (p = .878)	18.04 ± .567 (N = 10) (p = .030)	18.96 ± 1.17 (N = 10) (p = .024)	18.38 ± .961 (N = 10) (p = .068)	18.67 ± 1.08 (N = 20) (p = .012)
GAPDH	20.79 ± .814 (N = 10) (p = .367)	21.45 ± 1.45 (N = 10) (p = .068)	19.86 ± .667 (N = 10) (p = .220)	21.14 ± 1.13 (N = 10) (p = .014)	20.50 ± 1.11 (N = 20) (p = .022)
HMBS	27.74 ± 1.29 (N = 10) (p = .855)	26.37 ± 1.49 (N = 10) (p = .411)	25.20 ± 1.04 (N = 10) (p = .655)	27.37 ± 1.34 (N = 10) (p = .681)	26.29 ± 1.62 (N = 20) (p = .835)
HPRT	26.58 ± .815 (N = 10) (p = .202)	27.32 ± .941 (N = 10) (p = .303)	26.50 ± .811 (N = 10) (p = .612)	25.89 ± .955 (N = 10) (p = .514)	26.19 ± .916 (N = 20) (p = .210)
B2M	17.22 ± 1.66 (N = 10) (p = .868)	17.32 ± 3.98 (N = 10) (p = .512)	16.35 ± 1.62 (N = 10) (p = .551)	16.78 ± 1.41 (N = 10) (p = .401)	16.57 ± 1.499 (N = 20) (p = .919)
IL-6	33.54 ± 1.05 (N = 10) (p = .402)	31.94 ± 3.44 (N = 10) (p = .573)	32.06 ± 2.48 (N = 10) (p = .665)	31.28 ± 1.70 (N = 10) (p = .624)	31.67 ± 2.112 (N = 20) (p = .254)

#### Determination of the stability of housekeeping genes by geNorm

**Fig. 1:**
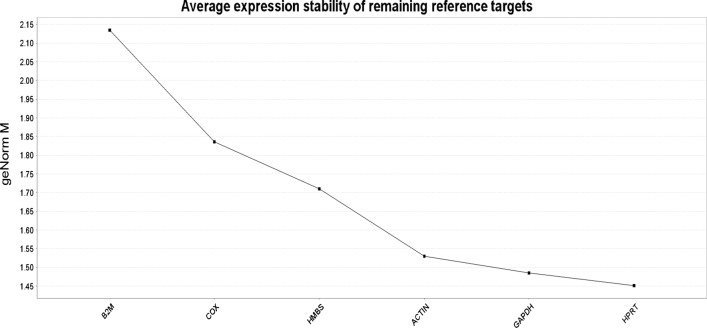
Determination of the stability of housekeeping genes by geNorm. Average stability value M is plotted for selected candidate reference genes. Here, lowest M value corresponds to the most stable gene

geNorm calculates the average pairwise variation between an individual gene and all other reference genes as the gene expression stability measure M. The gene with the highest M value is considered the least stable and usually M < .5 is suggested as a cut-off limit of variability. Thus, a gene with M value < .5 should be considered as a reliable stable reference gene. In present study, HPRT was found to be the most stable gene, followed by GAPDH and ACTB by geNorm (Fig. 1).

#### Determination of the stability of housekeeping genes by Normfinder

**Fig. 2:**
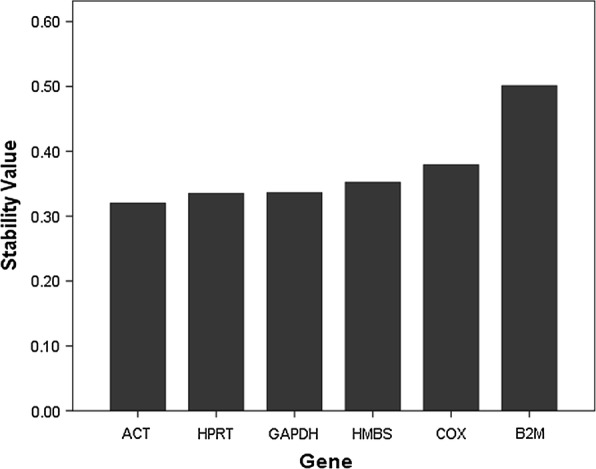
Determination of the stability of housekeeping genes by Normfinder. The stability value obtained by Normfinder is plotted against the genes. Here, the gene with the highest stability value is the least stable gene

Normfinder ranks reference genes according to their expression stability with the gene having the smallest stability value as the top ranked gene. In our study, out of the six chosen genes, Normfinder showed ACTB as the best reference gene with a stability value of .320 and combination of ACTB and GAPDH with a stability value of .236. Followed by ACTB in the ranking was HPRT, GAPDH, HMBS, COX and B2M (Fig. 2).

#### Determination of optimal number of housekeeping genes

According to the MIQE guidelines, normalization using a combination of genes as opposed to a single reference gene would yield more reliable results. geNorm calculates the pairwise variation Vn/Vn + 1 between two sequential normalization factors containing an increasing number of genes to determine the effect of addition of the next gene to the normalization factor. The program suggests use of all the six reference genes for normalization as all the Vn/Vn + 1 values are well above the .15 threshold suggested by the program (Additional file 7: Figure S6).

#### Effect of normalization strategies on selected target gene expression (IL-6)

The study was designed based on previous reports [9, 17]. For determining the best normalization strategy, gene expression analysis against target gene IL-6 was studied. Based on the result obtained for optimal number of reference genes, two normalization strategies were adapted (a) normalizing to geometric mean of the three most stable reference genes ACTB, HPRT and GAPDH, combinations based on top ranks geNorm and Normfinder (b) normalizing to geometric mean of three least stable genes COX, B2M and HMBS. In both the strategies, significant upregulation of IL-6 expression was confirmed. The fold changes obtained using stable genes showed a consistent rise in all the groups with values of .32, .52, .69, and .62 in HC vs. OFI, NSD, SD and All Dengue groups respectively. The fold changes obtained using unstable genes showed an inconsistent pattern with values of .49, .46, 2.12, and 1.10 in HC vs. OFI, NSD, SD and All Dengue groups respectively.

### Discussion

qPCR is a robust method used frequently for accurate gene expression analysis. However, if the data is not normalized to the most stable genes, the results could be highly misleading. In order to avoid this, it is important to use the best methods for normalization of Cq values. In our study, we adopted genetic normalization method’s geNorm and Normfinder programs to find the best-fit reference gene from COX, ACTB, GAPDH, HMBS, and HPRT and B2M to study the human gene expression during dengue viral infection. The Cq values generated for all the candidate reference genes were consistent with standard reference values.

Out of the 6 genes studied, HPRT was the most stable gene followed by GAPDH and ACTB according to geNorm analysis while Normfinder suggested ACTB as the most stable gene followed by HPRT and GAPDH. On the other hand, HMBS, COX and B2M were reported to be the least stable genes by both the programs in the same order with B2M being the least stable among the six reference genes. The ranking of the most stable genes was not in the same order in both the programs, which is probably because of the different algorithms employed by the programs. The result for finding the optimal number of reference genes by geNorm suggested the use of all the six genes in order to normalize effectively since the values for pairwise variation were all greater than the threshold value of .15. This threshold value is only a suggestion and not recommended as a strict cut-off by the program itself. The best strategy however is the use of at least three reference genes in order to achieve a more accurate and reliable normalization [11, 12].

In order to determine if the combination suggested by the programs was accurate, the relative fold expression of the reference genes against IL-6, an anti-inflammatory cytokine produced during dengue was studied by normalizing to the geometric mean of the reference genes in two groups (i) most stable genes (ACTB, HPRT, GAPDH) and (ii) least stable genes (COX, B2M, HMBS). The IL-6 gene has been reported as showing an increase to marked increase from the NSD to SD group in previous studies [4, 13]. In present study, the gene expression in terms of fold change was assessed for both the groups and is shown in Additional file 8: Figure S7. Both the strategies showed a consistent upregulation in fold expression. However, the poorly performing combination of COX, B2M and HMBS showed an inconsistent expression with a 2.12-fold increase in SD cases and 1.12-folds increase in All Dengue cases. Although the ranking of the most stable genes is in geNorm (HPRT>APDH>ACTB) is different from Normfinder (ACTB>GAPDH>HPRT), we would like to recommend the use of ACTB along with HPRT or GAPDH since the use of both has been supported with enough evidence in literature. Analogous to the present study, ACTB, HPRT and GAPDH have been found to be good reference genes from PBMC earlier as well. HPRT has been used as a single reference gene for dengue studies [14, 15]. Similarly, ACTB has also been widely used in dengue related studies [16, 17] while GAPDH has been used in dengue studies as sole reference gene or with combination of other reference genes [18, 19]. The other genes selected namely COX and B2M are found to be upregulated [20, 21] while HMBS is downregulated in dengue [22], this differential expression of these genes during dengue infection eliminates their use as a candidate reference gene, supporting the findings of the present study.

### Conclusion

To the best of our knowledge, this is one of the first report on the evaluation of candidate reference gene for studying host-responsive gene expression in dengue cases. Out of six endogenous genes, HPRT, ACTB, and GAPDH were found to be the stable set of candidate reference genes. Normalization by taking geometric mean of these three genes can be used for determining accurate fold change expression of host responsive genes in dengue virus infected cases. For most reliable normalization with two genes, we would suggest a combination of ACTB along with HPRT or GAPDH in dengue infection cases.

## Limitation

Less number of samples used in the study to find the relative fold change of IL-6 transcript expression in severe and non severe groups may be considered as a short coming of the present study.

## Additional files

**Additional file 1: Table S1.** Candidate reference genes evaluated in this study and its primers.

**Additional file 2: Figure S1.** Gel image of ACTB.

**Additional file 3: Figure S2.** Gel image of COX and IL-6.

**Additional file 4: Figure S3.** Gel image of GAPDH.

**Additional file 5: Figure S4.** Gel image of HMBS and HPRT.

**Additional file 6: Figure S5.** Gel image of B2M and IL-6.

**Additional file 7: Figure S6.** Determination of optimal number of housekeeping genes using geNorm. geNorm calculates pair-wise variation (Vn/n + 1) analysis between the normalization factors NFn and NFn + 1 to determine the optimal number of reference genes required.

**Additional file 8: Figure S7.** Validation of normalization strategies. For determining the best normalization strategy, the gene expression of IL-6 in terms of fold change is studied by comparing the normalization of IL-6 to the geometric mean of two groups (i) three most stable gene (ACTB_GAPDH_HPRT) and (ii) three least stable genes (B2M_COX_HMBS). Results are expressed as mean fold change and statistical significance was estimated using Shapiro–Wilk’s test. Different fold changes within the same target gene thus obtained are due to the different normalization strategies only. Here asterisks indicate p < .05. *ns* not significant.

## Abbreviations

DENV: dengue virus
DF: dengue fever
DHF: dengue haemorrhagic fever
DSS: dengue shock syndrome
qPCR: quantitative polymerase chain reaction
COX: cyclooxygenase
ACTB: β-actin
GAPDH: glyceraldehyde-3-phosphate dehydrogenase
HMBS: hydroxymethyl-bilane synthase
HPRT: hypoxanthine phosphoribosyl-transferase
B2M: β-2-microglobulin
IL: interleukin
TNFα: transforming factor α
INF γ: interferon γ
HKG: housekeeping genes
MIQE: *M*inimum *I*nformation for Publication of *Q*uantitative Real-Time PCR *E*xperiments
OFI: other febrile illness
NSD: non severe dengue
SD: severe dengue
HC: healthy control
