# Supplementary material for: Endogenous gene selection for relative quantification PCR and IL6 transcript levels in the PBMC’s of severe and non-severe dengue cases
Source: BMC Res Notes. 2018 Aug 2;11:550. doi: 10.1186/s13104-018-3620-2 (PMC6071388; doi:10.1186/s13104-018-3620-2)
Supplement: Supplementary file 1 — Additional file 1: Table S1. Candidate reference genes evaluated in this study and its primers. [file 13104_2018_3620_MOESM1_ESM.pdf]

Table S1

Candidate referen ce genes evaluated in this study and its primers

Symbol	Name	Forward Primer	Reverse Primer
**COX**	cyclooxygenase	AGCAGCTTTTCCAGACGACC	CGGTTGCGGTATTGGAACTG
**ACTB**	β- actin	AGAGCTACGAGCTGCCTGAC	AGCACTGTGTTGGCGTACAG
**GAPDH**	glyceraldehyde-3-phosphate dehydro-genase	AGGGCTGCTTTTAACTCTGGT	CCCCACTTGATTTTGGAGGGA
**HMBS**	hydroxymethyl-bilane synthase	TGCAACGGCGGAAGAAAA	ACGAGGCTTTCAATGTTGCC
**HPRT**	hypoxanthine phosphoribosyl-transferase	CTGGCGTCGTGATTAGTGAT	CTCGAGCAAGACGTTCAGTC
**B2M**	β-2-microglobulin	CTCCGTGGCCTTAGCTGTG	TTTGGAGTACGCTGGATAGCCT
**IL-6**	interleukin 6	AATGAGGAGACTTGCCTGGT	GCAGGAACTGGATCAGGACT
