# Supplementary figures and images for: Endogenous gene selection for relative quantification PCR and IL6 transcript levels in the PBMC’s of severe and non-severe dengue cases
Source: BMC Res Notes. 2018 Aug 2;11:550. doi: 10.1186/s13104-018-3620-2 (PMC6071388; doi:10.1186/s13104-018-3620-2)

Figure S1

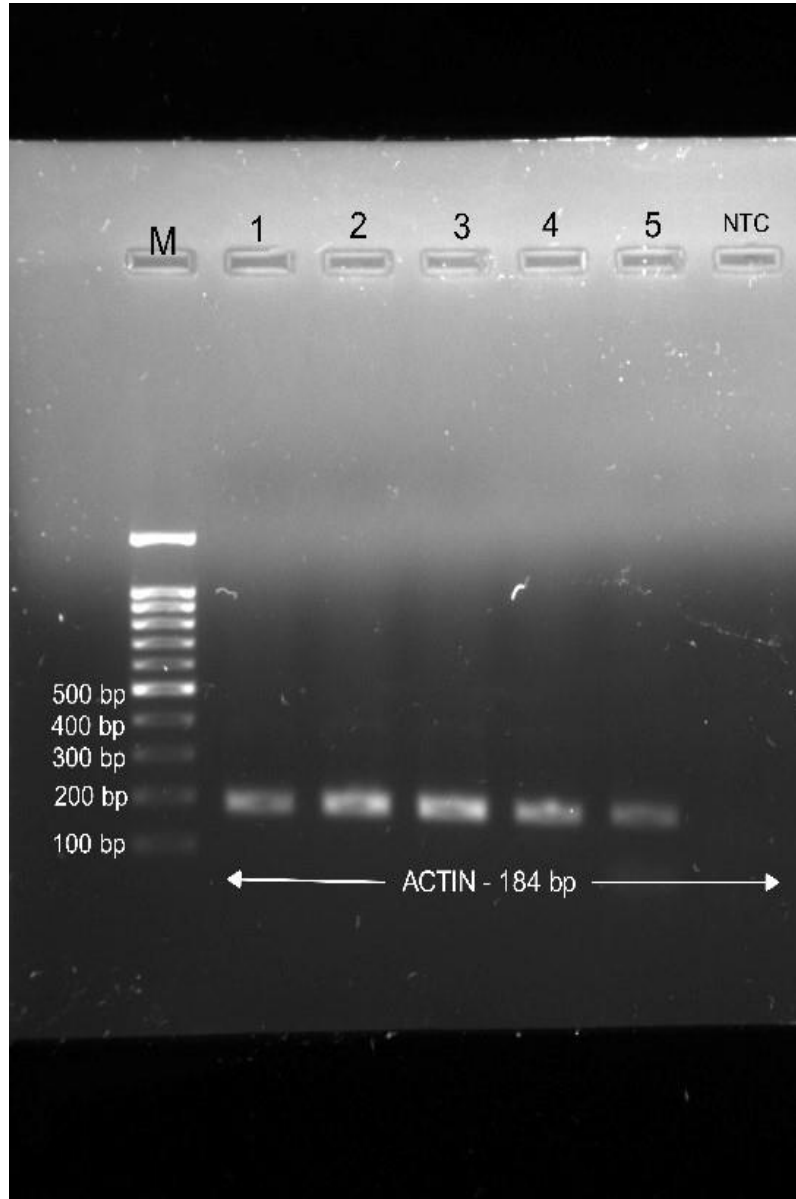

Gel image of *ACTB*

Supplement: Supplementary file 2 — Additional file 2: Figure S1. Gel image of ACTB. [file 13104_2018_3620_MOESM2_ESM.pdf]

Figure S2

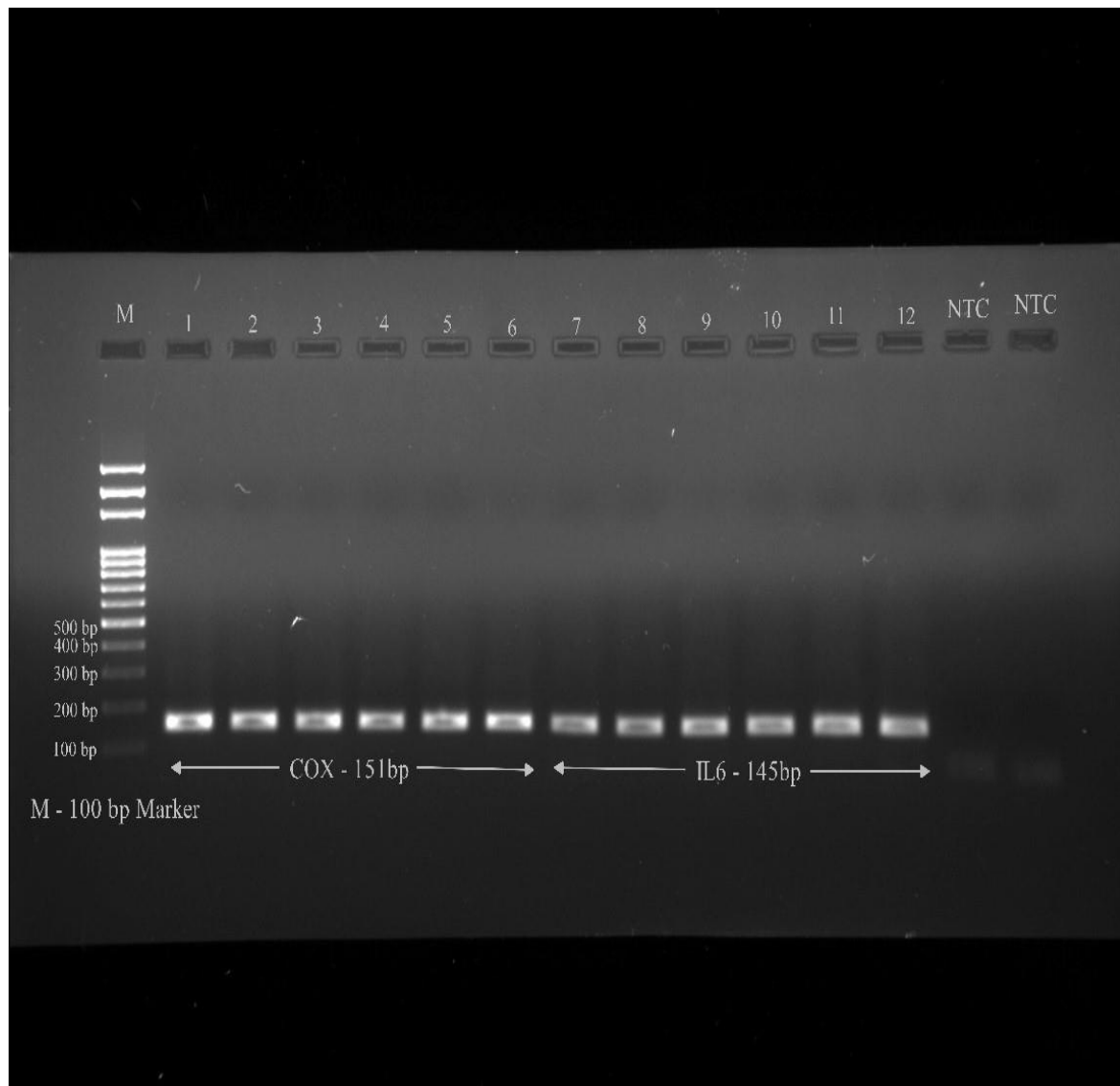

Gel image of *COX* and *IL-6*

Supplement: Supplementary file 3 — Additional file 3: Figure S2. Gel image of COX and IL-6. [file 13104_2018_3620_MOESM3_ESM.pdf]

Figure S3

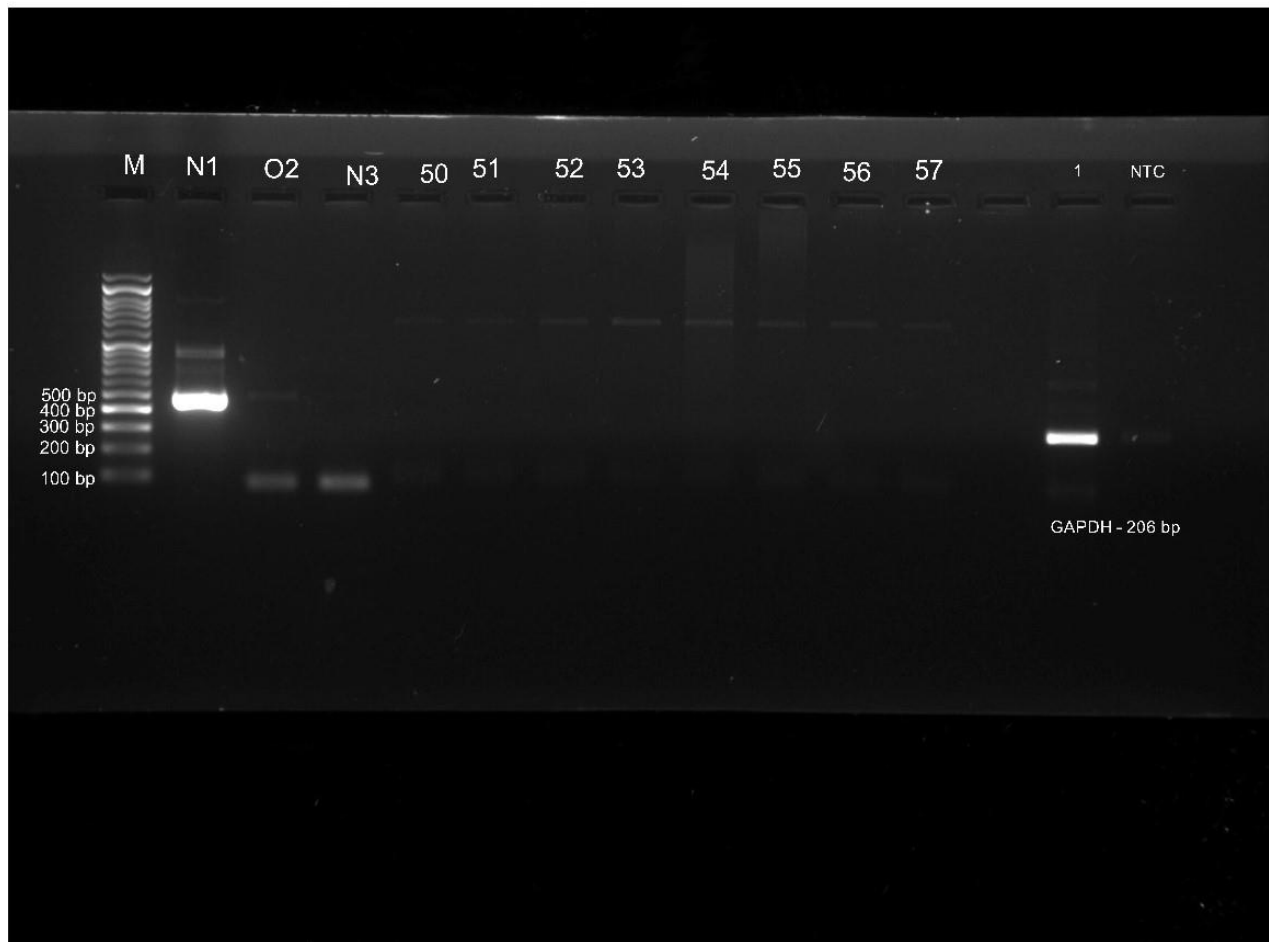

Gel image of *GAPDH*

Supplement: Supplementary file 4 — Additional file 4: Figure S3. Gel image of GAPDH. [file 13104_2018_3620_MOESM4_ESM.pdf]

Figure S4

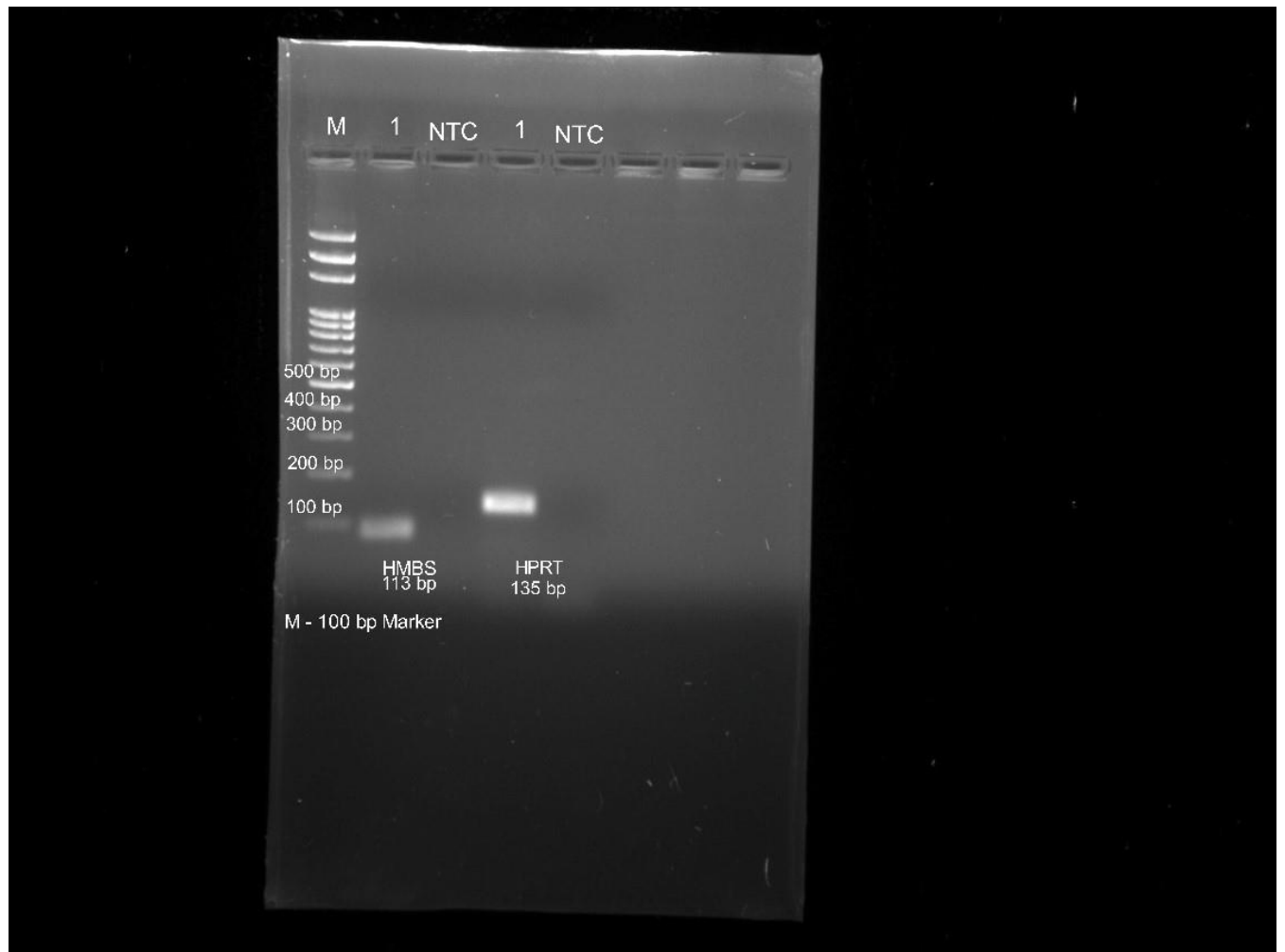

Gel image of *HMBS* and *HPRT*

Supplement: Supplementary file 5 — Additional file 5: Figure S4. Gel image of HMBS and HPRT. [file 13104_2018_3620_MOESM5_ESM.pdf]

Figure S5

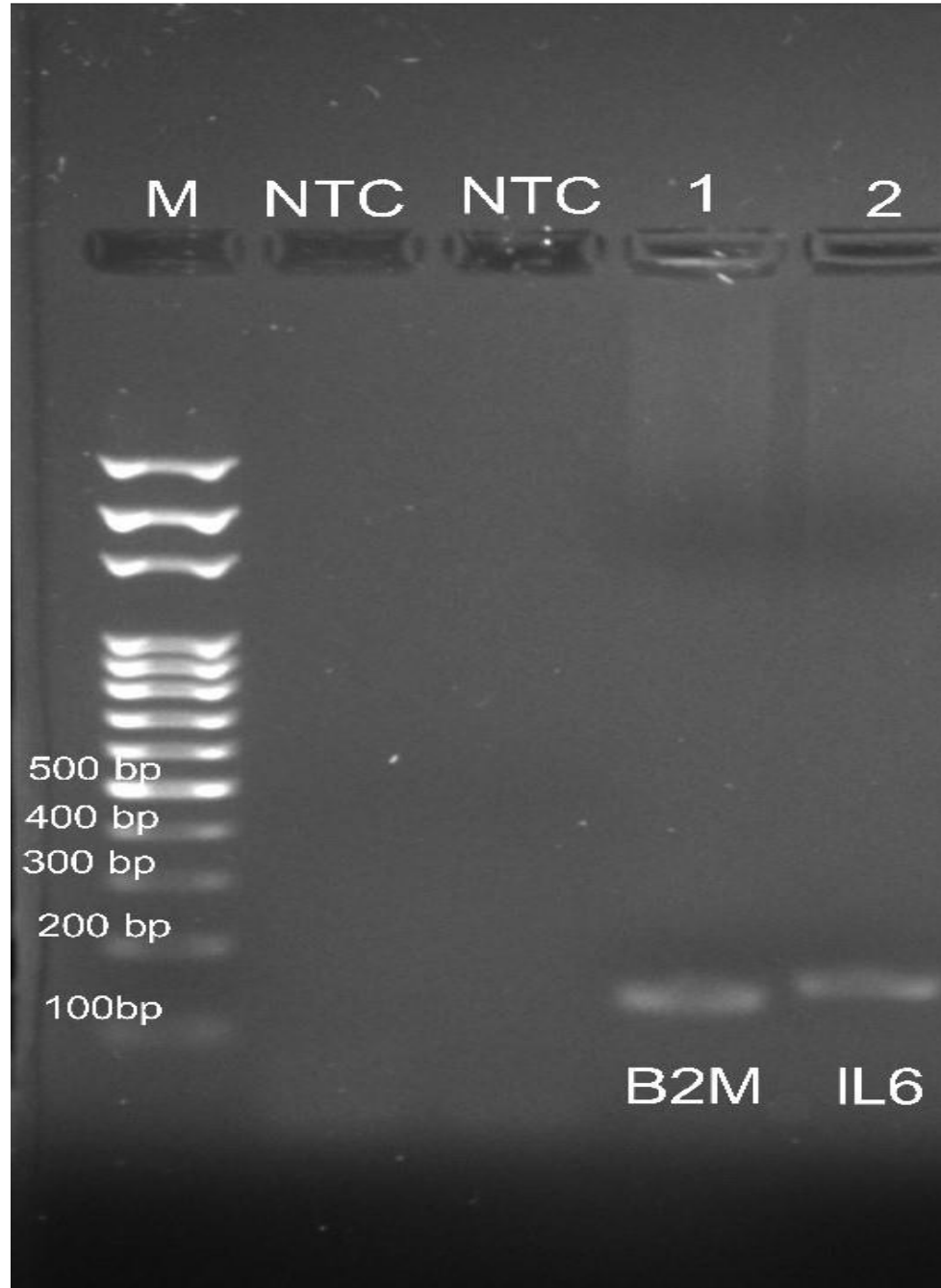

Gel image of *B2M* and *IL-6*

Supplement: Supplementary file 6 — Additional file 6: Figure S5. Gel image of B2M and IL-6. [file 13104_2018_3620_MOESM6_ESM.pdf]

Figure S6

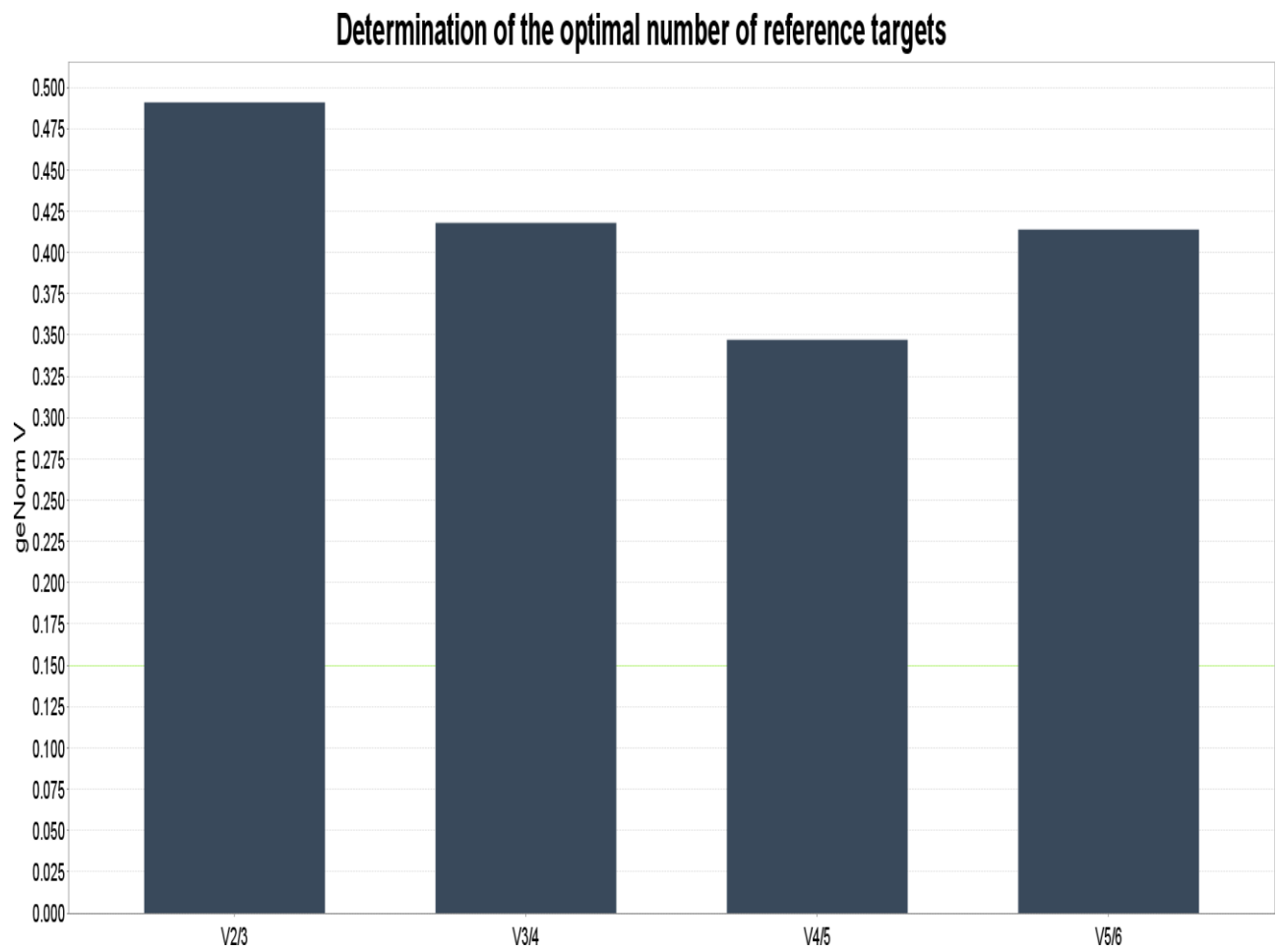

Supplement: Supplementary file 7 — Additional file 7: Figure S6. Determination of optimal number of housekeeping genes using geNorm. geNorm calculates pair-wise variation (Vn/n + 1) analysis between the normalization factors NFn and NFn + 1 to determine the optimal number of reference genes required. [file 13104_2018_3620_MOESM7_ESM.pdf]

Figure S7

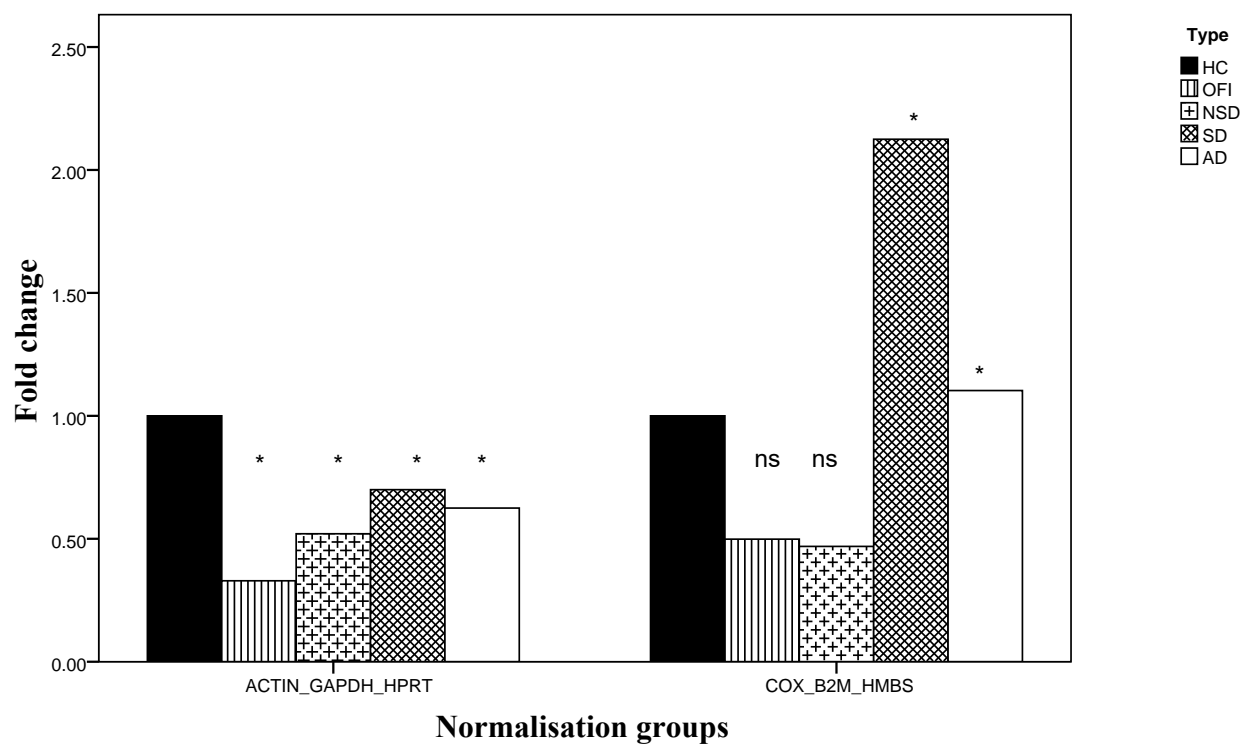

Supplement: Supplementary file 8 — Additional file 8: Figure S7. Validation of normalization strategies. For determining the best normalization strategy, the gene expression of IL-6 in terms of fold change is studied by comparing the normalization of IL-6 to the geometric mean of two groups (i) three most stable gene (ACTB_GAPDH_HPRT) and (ii) three least stable genes (B2M_COX_HMBS). Results are expressed as mean fold change and statistical significance was estimated using Shapiro–Wilk’s test. Different fold changes within the same target gene thus obtained are due to the different normalization strategies only. Here asterisks indicate p < .05. ns not significant. [file 13104_2018_3620_MOESM8_ESM.pdf]
